# A National and International Analysis of Changing Forest Density

**DOI:** 10.1371/journal.pone.0019577

**Published:** 2011-05-06

**Authors:** Aapo Rautiainen, Iddo Wernick, Paul E. Waggoner, Jesse H. Ausubel, Pekka E. Kauppi

**Affiliations:** 1 Faculty of Biosciences, University of Helsinki, Helsinki, Finland; 2 Program for the Human Environment, The Rockefeller University, New York, New York, United States of America; 3 Department of Forestry and Horticulture, Connecticut Experimental Agricultural Station, New Haven, Connecticut, United States of America; Colorado State University, United States of America

## Abstract

Like cities, forests grow by spreading out or by growing denser. Both inventories taken steadily by a single nation and other inventories gathered recently from many nations by the United Nations confirm the asynchronous effects of changing area and of density or volume per hectare. United States forests spread little after 1953, while growing density per hectare increased national volume and thus sequestered carbon. The 2010 United Nations appraisal of global forests during the briefer span of two decades after 1990 reveals a similar pattern: A slowing decline of area with growing volume means growing density in 68 nations encompassing 72% of reported global forest land and 68% of reported global carbon mass. To summarize, the nations were placed in 5 regions named for continents. During 1990–2010 national density grew unevenly, but nevertheless grew in all regions. Growing density was responsible for substantially increasing sequestered carbon in the European and North American regions, despite smaller changes in area. Density nudged upward in the African and South American regions as area loss outstripped the loss of carbon. For the Asian region, density grew in the first decade and fell slightly in the second as forest area expanded. The different courses of area and density disqualify area as a proxy for volume and carbon. Applying forestry methods traditionally used to measure timber volumes still offers a necessary route to measuring carbon stocks. With little expansion of forest area, managing for timber growth and density offered a way to increase carbon stocks.

## Introduction

Measuring forests tells their spatial extent and the density of the trees that occupy that extent. Traditionally foresters measured the attribute of merchantable timber, also called growing stock, on a given stand to assess its commercial value. As concerns expand from timber volume to include the attributes of biomass as well as the carbon sequestered in that mass, the attributes must be connected and new coefficients measured.

The Forest Identity [1] connecting those attributes shows timber volume equals area times density, and biomass equals volume times the ratio of growing stock volume to the biomass of crown, foliage, and roots. Finally, carbon mass equals the biomass times its carbon concentration.

Both nature and humanity affect forest area. While climate and geography determine potential forest area, humans determine the hectares they spare from farms, logging, settlement, and transportation.

As for density in a natural forest, climate and geography also affect forest productivity measured as cubic meters of timber growth per hectare. Humans may degrade forests and deplete their timber, biomass, and carbon, or they can manage them by planting faster growing trees, improving sites, and sparing mature and dead trees [2], [3], [4]. Because trees grow for decades, the resulting rise in density becomes apparent decades after degradation. Managing intensively, humans can take advantage of techniques developed to speed growth and increase density used in tree plantations [5].

Here we examine the effectiveness of changing forest area and density to change timber volume and carbon in a nation with a continuing forest inventory and in a global inventory of nations encompassing a little over two thirds of global forests. We begin with the inventory of a single nation that avoids some discrepancies caused by national differences in method, continuity, and capacity [6], [7]. We then proceed to a less certain global inventory.

## Results

### Forests in a single nation with continuing inventory

The United States represents a single nation with a continuing inventory. We examined measurements from 1953 to 2007 by the United States Forest Service (USFS) [8]. The USFS has published estimates of forest area, timberland area, and growing stock on timberland using a standard, continuing system. Timberland, which comprises about two-thirds of U. S. forest area, is not legally reserved from timber harvest and is capable of annually increasing density per hectare by a minimum of 1.4 cubic meters of industrial wood.

**Figure 1:**
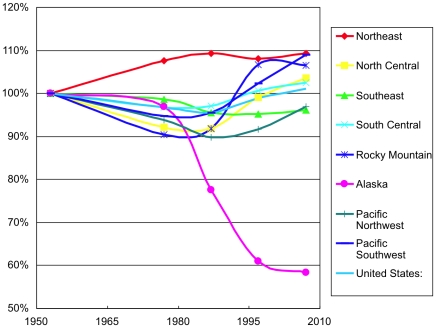
United States timberland area by region, 1953–2007. The conservation measure of reclassifying large areas as non-forest or wilderness caused the exceptional case of declining timberland in Alaska. Index 1953 = 100%.

In US regions since 1953, timberland area generally changed little, with excursions up and down always less than 10%, Figure 1. Overall timberland area grew 1% between 1953 and 2007 in the United States. Total forest land in the United States rose by 0.5% over the same period.

**Figure 2:**
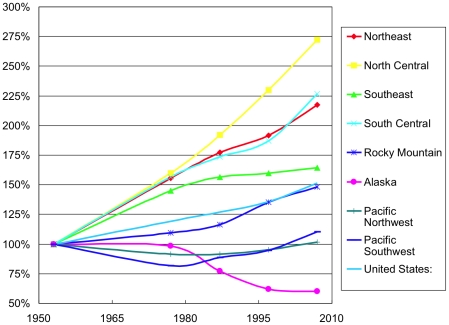
United States volume of growing stock on timberland by region 1953–2007. The conservation measure of reclassifying large areas as non-forest or wilderness caused the exceptional case of declining volume in Alaska. Index 1953 = 100%.

During the same years, the volume of growing stock on the same land generally rose. Demonstrating the greater change in volume than area, the vertical axis for charting volume in Figure 2 extends several times that for charting area in Figure 1. In the two North and the South Central regions, volume rose sharply during a decades-long restoration [1]. In the Southeast, the restoration of volume that began in the 19^th^ century continued but slowed. In the Pacific regions, volume recovered from a dip in the 1980s. All in all, the combined national volume swelled 51%. Clearly, growing timber volume on a stable area indicates growing density.

**Figure 3:**
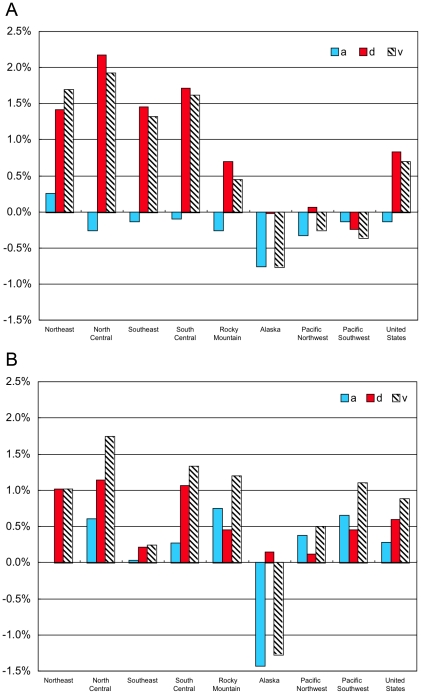
Annual change in timberland area *a*, density *d*, and timberland volume *v* in the United States (a) 1953–1987 (b) 1987–2007.

The Forest Identity shows volume, *V* equals the area, *A* times density, *D* or *V* = *D*×*A*. We denote the annual % changes in volume, area, and density using small case letters *v*, *a*, and *d*. Because the annual percentage changes are small, it can be shown that the sum of changing area *a* and density *d* nearly equals changing volume *v = a+d* (see Materials and Methods section). Figures 3a–b show the changing area, *a* and density, *d* and the consequent changing volume, *v* during 1953–1987 and during 1987–2007. These two periods were selected based on the available data for the year 1953 and decadal data for 1977–2007. In the first period in the two Eastern, two Central, and the Rocky Mountain regions, growing density increased volume by overwhelming small area changes. In the Western regions and Alaska, losses in area combined with changes in density to shrink volume. For the United States as a whole during the first period, the average annual 0.83% more density less 0.13%/yr less area increased average volume 0.70%/yr.

During 1987–2007, density continued to rise in the Eastern, Central, and Rocky Mountain regions, though at a slower pace. Volume fell in Alaska and the western regions despite small density gains in Alaska and the Pacific Northwest and a large area addition in the Pacific Southwest. For the United States as a whole during the second period, the average annual 0.60% more density plus an additional 0.28%/yr area increased average volume by 0.88%/yr.

### International Forests

For a broader understanding of changing area and density, we analyzed international data compiled by the United Nations Food and Agriculture Organization (UNFAO) in the 2010 Global Forest Resources Assessment [9]. Difficulties creating reliable time series from UNFAO reports stem from 1) inconsistent reporting criteria and data quality from member countries, 2) frequent retroactive revisions by the UNFAO, and 3) changing definitions of forest attributes [6]. To address these problems our analysis relies on the latest 2010 publication, which provides a consistent data series for the years 1990–2010.

The UNFAO reports forest area rather than timberland. Because the 2010 report published sequestered carbon but not growing stock volume for 1990–2010, we analyzed carbon rather than volume. Sequestered carbon is the product of volume and two variables: the ratio of biomass to volume and of the concentration of carbon in the biomass. If the ratio and concentration are nearly constant during 1990–2010, the annual percentage change of carbon that we present nearly equals the change of growing stock volume.

Countries meeting data quality criteria were included in the analysis as described in the Materials and Methods section. Table S1 provides a list of the 68 countries included in this analysis by region. These countries provided a global sample that accounted for 72% of the reported global forest area and 68% of the reported global carbon. The following summarizes global results by placing the 68 countries in the 5 regions named for continents as listed in Table S1.

Only 10 countries in the continent of Africa met the quality criteria. 80% of the forest area and carbon mass in the Russian Federation were included in the Asia region and 20% in the Europe region roughly corresponding to the share of each [10]. Australia was included in the Asian region, where it accounted for 10% of total forest carbon in that region.

**Figure 4:**
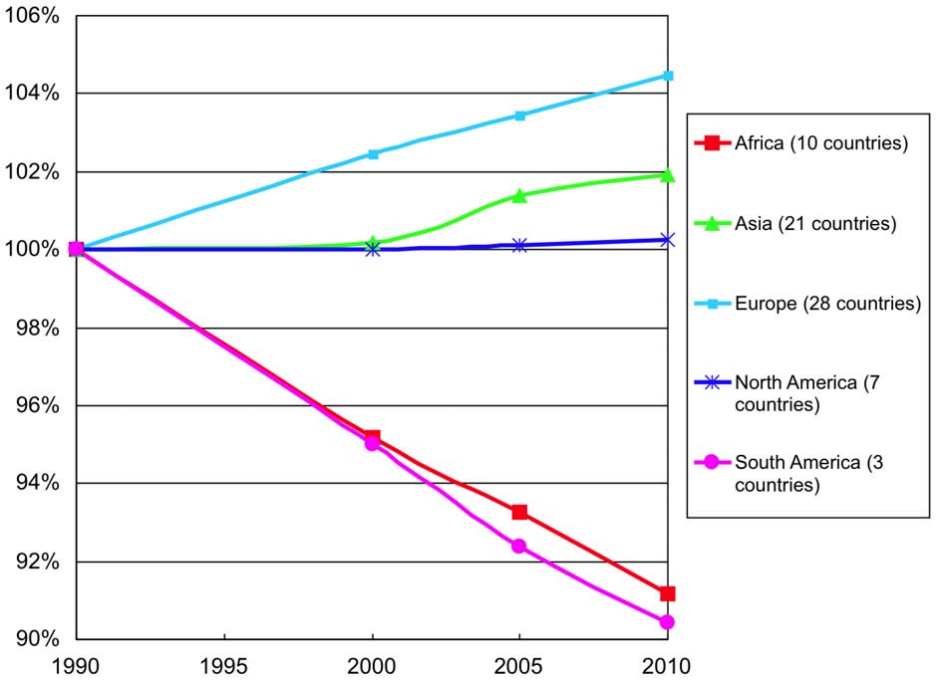
Forest area by region 1990–2010.

Countries in the South American and African regions lost close to 10% of their forest area during the two decades, Figure 4. Asian and European forest area expanded several percent while the area in North America changed little.

**Figure 5:**
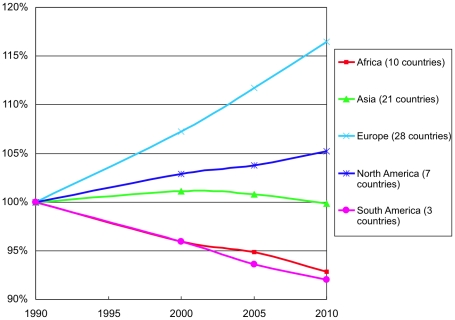
Carbon mass by region 1990–2010.

The changing carbon mass graphed in Figure 5 combined with the area reflects changing density. During the second of the two decades, carbon mass in the Asian region changed little, while area expanded, indicating falling carbon density. For analyzed countries in the Africa and South America regions, carbon declined slightly less than area, reflecting small density increases. North America and Europe gained carbon well in excess of any area additions.

**Figure 6:**
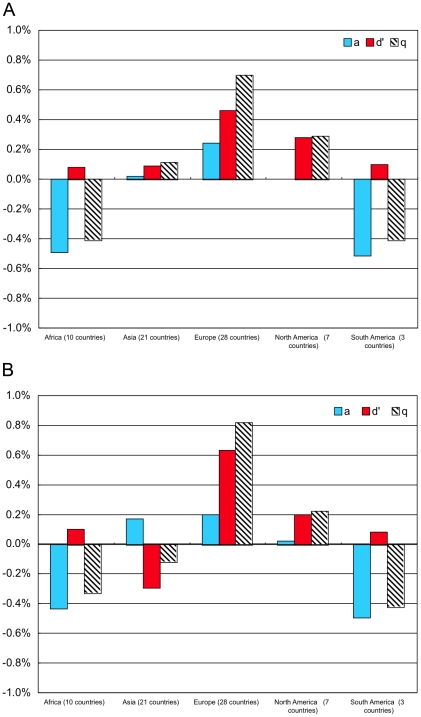
Annual change in forest area *a*, carbon density *d′*, and carbon mass *q* by region (a) 1990–2000 (b) 2000–2010.

The Forest Identity shows the mass of sequestered carbon, *Q* equals the area, *A* times the density, *D′* of carbon per area or *Q = D′×A*. Because the annual percentage changes denoted by lower case letters are small, the changing area, *a* plus changing carbon density, *d′* nearly equals changing mass *q = a+d′* (see Materials and Methods section). Figures 6a–b show the changing area *a* and density *d′* and the consequent changing mass *q* for two decades, 1990–2000 and 2000–2010. This analysis parallels that of U.S. timber volume, replacing changes in timber density and volume with changes in carbon density, *d*′ and carbon mass, *q′*.

During 1990–2000 carbon density grew in all regions. While area changed little in the Asian and North American regions, rising density increased sequestered carbon. In the European region, increasing area plus carbon density together grew the mass of carbon. The data for the African and South American regions indicate shrinking carbon volume but slight gains in carbon density during the 1990s.

During the second period, 2000–2010, the Asian region displayed the greatly altered pattern. The great loss of density and sequestered carbon in Indonesia obscured the rising density in ten of the twenty-one nations included in the region. As during the preceding period, rising density contributed most to increased sequestered carbon in the European and North American regions. African and South American losses of sequestered carbon mass were again tempered by slightly rising carbon density.

**Figure 7:**
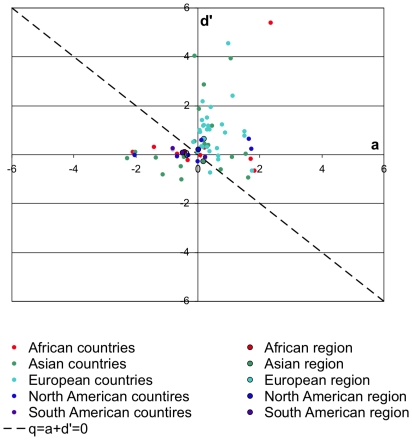
Annual change in forest area *a* and carbon density *d′* 2000–2010. The forest in countries above the diagonal line (v = a+d′ = 0) gained carbon mass, while countries below it lost carbon mass.

In all regions apart from Asia, the sign of change in forest area and the stock of sequestered carbon was the same. However, their magnitude differed significantly, especially in Europe and North America where most of the change was attributable to increasing carbon density (Figs. 6a and 6b). As *q = a+d′*, area is a suitable proxy for forest carbon only if carbon density remains constant (*d′* = 0). The discrepancy between the rates of area and density change becomes even more apparent, when the combinations of *a* and *d′* are examined for individual countries instead of regional aggregates (Figure 7). Forest area and carbon changed in tandem only in those countries whose points in Figure 7 fall on the diagonal axis.

## Materials and Methods

Data for the national analysis for forests in the United States come from the 2009 edition of Forest Resources of the United States, 2007 [8] published by the US Forest Service. International data come from the 2010 Global Forest Resources Assessment [9] published by the UNFAO.

We relied on the Forest Identity [1], which equates growing stock density *D* = *V/A* to the volume *V* and area *A*, and equates carbon density *D′* = *Q/A* to the mass of carbon *Q* per area *A*.

To estimate annual changes denoted by small case letters, we use the convention *x* = ln(*X_2_/X_1_)/(f−i*) where *X_f_* and *X_i_* denote the value of *X* for final and initial year of the period being analyzed and (*f−i*) is the number of years in the interval. This operation is justified for small changes i.e., Δ*X*≪*X*. Doing so, we get the equation *v = a+d* for changing timber volume and *q = a+d′* for changing carbon mass.

The 68 nations for analysis were selected from the 2010 Global Forest Resources Assessment by the following criteria:

- Data on carbon mass *Q* must be available for the years 1990, 2000, 2010.
- Carbon density *Q/A* must have changed sufficiently (≥0.1 tons/ha) during both periods (1990–2000, 2000–2010) to avoid nations where *Q* was likely extrapolated using a constant *Q/A* ratio.
- Carbon mass *Q* must have changed sufficiently (≥0.1%) during both periods to avoid nations that reported constant *Q* for all years.
- The rate of change of *Q* must have changed sufficiently during both periods to avoid countries that linearly extrapolated or/interpolated *Q* for all years.

For borderline cases, we examined national reports and made some exceptions based on the description of their methods. Table S1 shows the nations analyzed.

## Discussion

Whether measured by area or carbon content, the countries included in this study encompass slightly over two thirds of the global forests, leaving one third of the planet's forests unaccounted for. Nonetheless, despite uncertainties, especially among international compilations of national forest inventories, a large principle emerges. Forest area and density change independently with consequences for timber volumes and carbon sequestration.

For measuring two-dimensional forest area, remote sensing (i.e., satellite monitoring) offers the best solution for comprehensive standardized data collection. Reduced grid sizes will offer even greater precision [11], [12]. Tests of the ground truth of satellite results for forest area yield results like those found in India [13] establishing accuracies as high as 96%.

For measuring three-dimensional volume, however, accurate estimates rely on ground level measures of the size distribution of trees. Without field measurement or another indication of tree size, implying forest health, or carbon storage, from area alone will continue to be highly uncertain and even misleading [14]. Remote sensing that incorporates estimation of forest height (i.e., LIDAR) as well as area offers promise for reducing the difficulties involved with collecting data on three dimensional as opposed to two dimensional attributes [15], [16]. Currently, forest inventories based on field measurements provide the most accurate appraisals of the development over time of timber volume and forest carbon, as well as the most sound basis for anticipating future inventories.

For forests in a world with a growing population, with growing needs, but a fixed expanse of land, faster increases of volume or carbon than decreases of area during recent decades are encouraging. Technological improvements have been shown to improve greatly the efficiency of producing commercial timber products using less land [17], [18]. The major stresses on forest land are thus attributable to humanity's appetite for forest land, not forest products.

To stop the loss of forest land in countries still experiencing deforestation, lifting crop yields as well as stabilizing population and switching fuels can reduce pressure on forest land [19], [20]. Forest management to increase carbon density by encouraging growth of young forests and improving degraded forests offer effective levers for higher global forest carbon sequestration.

Inventories taken steadily by a single nation, the United States, and other inventories gathered recently from many nations by the United Nations confirm the asynchronous effects of changing area and density. While assessing the environmental impacts from forest management has become integral to national environmental policies and the global debate over climate change, current global forest statistics continue to suffer from deficiencies in the availability, accuracy, and precision of measurements of key attributes. The need to address these issues with scientific rigor calls for increased attention to forest density measurement to produce a consistent global data set that acknowledges the importance of forest density, in addition to area, as a decisive factor.

## Supporting Information

Table S1 List of countries for international analysis.
(DOC)

## References

[1] Kauppi PE, Ausubel JH, Fang J, Mather AS, Sedjo RA (2006). Returning forests analyzed with the forest identity.. Proc Natl Acad Sci USA.

[2] Sedjo RA, Kallio M, Dykstra DP, Binkley CS (1987). Forest resources of the world: forests in transition.. The global forest sector: an analytical perspective.

[3] Sedjo RA (1984). An economic assessment of industrial forest plantations.. Forest Ecology and Management.

[4] Sedjo RA, Kallio M, Andersson AE, Seppälä R, Morgan A (1986). Forest plantations in the tropics and southern hemisphere and their implications for the economics of temperate climate forestry.. Systems analysis in forestry and forest industries.

[5] Kirilenko AP, Sedjo RA (2007). Climate change impacts on forestry.. Proc Natl Acad Sci USA.

[6] Grainger A (2008). Difficulties in tracking the long-term global trend in tropical forest area.. Proc Natl Acad Sci USA.

[7] Waggoner PE (2009). Forest inventories. Discrepancies and uncertainties.. http://www.rff.org/RFF/Documents/RFF-DP-09-29.pdf.

[8] Smith WB, Miles PD, Perry CH, Pugh SA (2009). Forest Resources of the United States, 2007. Gen. Tech. Rep. WO-78.

[9] United Nations Food and Agriculture Organization (2010). Global Forest Resources Assessment 2010.. http://www.fao.org/forestry/fra/en/.

[10] UNECE/UNFAO (2003). http://www.unece.org/timber/docs/dp/dp-27.pdf.

[11] Sánchez-Azofeifa GA, Castro-Esau KL, Kurz WA, Joyce A (2009). Monitoring carbon stocks in the tropics and the remote sensing operational limitations: From local to regional projects.. Ecological Applications.

[12] Kalacskaa M, Sanchez-Azofeifaa GA, Rivarda B, Calvo-Alvaradob JC, Quesadac M (2007). Baseline assessment for environmental services payments from satellite imagery: A case study from Costa Rica and Mexico.. Journal of Environmental Management.

[13] Forest Survey of India, Ministry of Environment and Forests (2008). State of Forest Report 2003..

[14] Wernick IK, Waggoner PE, Kauppi PE, Sedjo RA, Ausubel JH (2010). Quantifying forest change.. Proc Natl Acad Sci USA.

[15] Næsset E, Gobakken T (2008). Estimates of above- and below-ground biomass across regions of the boreal forest zone using airborne laser.. Remote Sensing of the Environment.

[16] Fagan M, DeFries R (2009). Measurement and Monitoring of the World's Forests: A Review and Summary of Remote Sensing Technical Capability, 2009–2015.. http://www.rff.org/Publications/Pages/PublicationDetails.aspx?PublicationID=20971.

[17] Wernick IK, Waggoner PE, Ausubel JH (2000). The forester's lever: industrial ecology and wood products.. Journal of Forestry.

[18] Ausubel JH, Waggoner PE (2008). Dematerialization: Variety, caution and persistence.. Proc Natl Acad Sci USA.

[19] Waggoner PE, Ausubel JH (2001). How Much Will Feeding More and Wealthier People Encroach on Forests?. Pop Dev Rev.

[20] Victor D, Ausubel JH (2000). Restoring the forests.. Foreign Affairs.

