# Supplementary material for: A National and International Analysis of Changing Forest Density
Source: PLoS One. 2011 May 6;6(5):e19577. doi: 10.1371/journal.pone.0019577 (PMC3089630; doi:10.1371/journal.pone.0019577)
Supplement: Table S1 — List of countries for international analysis. (DOC) [file pone.0019577.s001.doc]

**Table S1**
